# Supplementary material for: Are carbon emissions trading and green financial instruments synergistic? -Comprehensive quantitative research based on content analysis
Source: PLoS One. 2024 Mar 7;19(3):e0298601. doi: 10.1371/journal.pone.0298601 (PMC10919601; doi:10.1371/journal.pone.0298601)
Supplement: S1 Data — (ZIP) [file pone.0298601.s001.zip › Data/地级市Z_F工作报告.zip!/╣π╢1⁄2/╟σ╘╢╩╨/└┤╫╘╣1⁄2╓┌║┼í╛┬φ┐╦╩2╛▌═°í┐_2019.docx]

各位代表：

我代表市人民政府，向大会作工作报告，请予审议，并请政协委员和其他列席人员提出意见。

2018年工作回顾

2018年是清远改革发展史上具有重大意义的一年，建市30周年，习近平总书记亲临视察。在省委、省政府和市委的正确领导下，我们全面贯彻习近平新时代中国特色社会主义思想，坚持新发展理念，聚焦高质量发展，全面深化改革，深入推进广清一体化、乡村振兴和区域协调发展，全力打造“两市两区”，强化民生保障，经济社会保持平稳健康发展，基本完成市七届人大三次会议确定的主要目标任务。发展呈现出新亮点，成功申办省第十六届运动会，清远国家高新区排位大幅提升9位，引进腾讯华南云计算基地项目，飞霞山市民公园建成，省级职教基地5所院校首期主体工程实现封顶，“高接高”、汕昆高速清远段、洲心大桥建成通车，广连高速全线动工，江南水厂通水试运行，7家农信社改制任务全部完成。

一年来，我们抓转型、优服务，发展质量效益稳步提升

经济运行总体平稳。预计全市完成生产总值1560亿元，比上年增长（以下简称增长）4%左右；完成固定资产投资760亿元，增长14%，其中重点项目完成年度投资111.6%；规上工业增加值442亿元，增长7.3%；社会消费品零售总额754亿元，增长10%；一般公共预算收入111.9亿元，增长8.6%；外贸进出口总额410亿元，增长25%；全社会用电量增长12.8%；12月末金融机构本外币存、贷款余额分别增长8%和13.7%。发展质量进一步提高，前11月规上工业经济效益综合指数提高20.4个百分点，利润总额增长22.1%；高技术制造业、先进制造业增加值分别增长12.5%和14.2%。非税比重降至27.9%。政府隐性债务压缩到可控范围。

转型升级扎实推进。加强招商引资，完善招商体制机制，筹建市招商中心，强化环保、效益和安全导向，举办面向高新、民营企业等招商活动，全市新签约工业项目109个，总投资536亿元；合同外资增长138%。出台工业技改三年行动实施方案，完成工业技改投资50亿元。新增规上工业企业7家。开展国际化旅游城市战略规划研究。清远长隆项目和周边配套建设有序推进。启动智慧新城、中以科技小镇建设。加快创建全域旅游示范区；新增4A级景区2个，总数达19个；接待游客4398.5万人次，实现收入351亿元，分别增长10.4%、11.6%。国企改革有序推进。电子商务较快发展。中心城区商业网点规划建设稳步推进。

创新发展步伐加快。首次获得国家科学技术奖。高新区排位增幅居全省首位。出台“创新十条”实施细则。大力培育创新企业和平台，新增高新企业83家、新型研发机构1家、省级企业技术中心12家、省级以上工程中心35家，实现国家小微企业双创示范基地、省重点实验室“零”的突破，华南863、天安智谷进驻项目405个。专利授权量、发明专利授权量分别增长83.2%和80.2%，新增国家知识产权优势企业9家。加快人才高地建设，校地合作深入推进，“海外院士工作站”启动，新增博士后科研工作站2个。成功举办众创杯、创业大赛、高端人才交流会等活动。

营商环境逐步优化。出台优化营商环境、提升开办企业便利度、鼓励建设使用标准厂房等政策，积极攻关营商环境关键制约问题。召开民营企业、拟上市企业、外贸企业座谈会，面对面帮助企业解决困难。落实各项降成本政策，为企业减负43.8亿元。深化“放管服”改革，加快“数字政府”建设，推进“证照分离”改革试点，建立粤东西北首个网上中介超市，强县放权115项，编制完成7043项政务服务事项标准，行政许可承诺时限比法定时限缩短50%以上，商事登记压减至2个工作日以内。全市新登记各类市场主体4万余户，企业户数增长12.9%。

一年来，我们抓共建、促平衡，区域协调发展迈出新步

一体化发展高质量推进。联合印发高质量推进广清一体化发展方案。成功举办粤港澳论坛及招商推介会。推进广清产业园A区扩园，新动工项目21个，累计签约项目179个，投（试）产项目36个。创新合作共赢模式建设广清产业园B区。两德合作区交接有序推进。广清农业众创空间、广百南部物流枢纽项目首期建成。推动广州银行设立清远分行。承接珠三角梯度转移项目60个，完成省定任务120%。全市园区完成规上工业增加值167亿元、全口径税收35.3亿元，分别增长12%和14%。206所学校、32家县级以上医院与广州建立了帮扶关系。

北部绿色发展加快。成功举办省第六届少数民族传统体育运动会。民族工业园升级为省级产业转移园。绿色能源、农业、旅游加快发展，桔子塘、雷公岩风电项目部分机组并网发电，华润风电福山、泉山项目进展顺利；阳山鸡、西洋菜、连山大肉姜成为地理标志产品，阳山成为国家生态原产地产品保护示范区；连南获评全国森林旅游示范县，鹿鸣小镇、三江花海等项目加快建设。德建水库完成主体工程，塘冲水库动工建设。连山、连南城镇特色化改造全面推进。

交通通达水平明显提升。出台中心区域道路交通三年行动计划，集中资源重点攻坚，完成道路建设改造项目30个。高速公路项目顺利推进，新增通车里程172公里，占全省1/4，总里程跃居全省第2位；佛清从高速前期工作取得积极进展。磁浮旅游专线首开段动工。广清城轨一期清远段完成轨道铺设。广清永高铁、广清地铁、佛江高速北延线规划衔接取得新进展。北江航道升级项目完成总投资68%。完成一批国省道改造项目。“四好农村路”建设加快，农村公路新硬化252公里。建成4G移动通信基站9847座，光纤入户92.4万户。

一年来，我们抓振兴、夯基础，农业农村发展活力增强

农综改稳步推进。成为省乡村振兴综合改革试点。“2+1+N”乡村振兴政策体系基本建立。“三个重心下移”“三个整合”深入推进，建立村民理事会1.7万个，村级公共服务站1200个；累计整合土地170.9万亩，占二轮承包耕地面积77.8%；整合涉农资金50.1亿元。农村金融改革加快，建立97家农村微合作金融组织，发放“两权”抵押贷款16亿元、银担支农贷款2.4亿元。完成农村土地承包经营权确权登记颁证。农村集体产权制度改革稳步推进。在全国首创“乡村新闻官”。

农业产业加快发展。按照乡村振兴“二十字”要求，谋划建设乡村振兴综合示范区。大力实施“3个三工程”，新增茶叶1.5万亩、柑橘2.8万亩、清远鸡1000万只。成功创建省级现代农业产业园4个。英德成为首批国家农村产业融合发展示范园创建单位。大力培育新型经营主体，新增省级农业龙头企业12家、农民合作社531家、家庭农场1201家。新增“三品一标”40个。清城区成立全省首家森林旅游专业合作社。乡村旅游蓬勃发展，成功承办广东人游乡村暨清远首届乡村旅游节、广东首届茶叶产业大会等活动。建成电商村级服务站450家。清新、英德成为省电商进农村综合示范县。积极有效应对非洲猪瘟疫情。

乡村环境更加宜居。出台“美丽乡村2025”行动计划，累计建成美丽乡村2357条，20户以上自然村82.2%完成人居环境综合整治，省定贫困村20户以上自然村90%完成“三清三拆三整治”。基本完成英德、连州、阳山省级新农村连片示范工程建设。“大棚房”整治积极开展。村庄规划覆盖率达34.3%。新增中国传统村落15个。完成中小河流治理184.8公里、高标准农田建设22.6万亩、农网改造投资6.2亿元。

脱贫攻坚扎实有效。出台脱贫攻坚三年行动计划。坚持精准施策，大力发展产业扶贫，“造血”与“输血”并重，预计可实现2.95万人预脱贫，累计实施产业扶贫项目8.8万个，带动贫困户3万户；建设光伏项目198个，带动贫困人口6万人；贫困就业转移3.1万人；发放扶贫贷款3.75亿元，规模居全省前列。4.4万无劳动能力贫困人口全部纳入政策保障，2.1万名建档立卡贫困学生教育补助全部落实。

一年来，我们抓生态、强治理，生态文明建设取得实效

百日攻坚成效明显。重点治理扬尘、工业企业、机动车、油烟和露天烧烤等大气污染问题，加大环境执法力度，全力改善空气质量，全市空气优良率比百日攻坚前提升10.3个百分点，全年空气质量综合指数同比改善2.2%。完成“煤改气”94条，淘汰10蒸吨及以下工业燃煤锅炉38台，完成33家省级VOCs重点监管企业治理，整治“散乱污”企业1445家。完成空气质量监测网络优化布点。

环保督察整改取得实效。全部办结中央、省环保督察交办的456宗案件，关停取缔企业81家，整改299家。实事求是加快推进4条黑臭水体整治，完成黄坑河、海仔大排坑城市建成区段整治任务，澜水河、龙沥大排坑整治加快。完成清新滨江迳口、连州龙潭寺取水口上移。完成芒洲饮用水源环境问题整治。全面推行河长（湖长）制，开展河道保洁专项行动，北江干流水质总体稳定，11个县级及以上集中饮用水源水质100%达标。整县推进污水处理设施建设加快。严厉打击非法转移处置固废行为。英德国家森林公园范围调整获批。

生态建设扎实推进。完成生态保护红线划定。启动创建国家森林城市。潖江蓄滞洪区前期工作基本完成。封山育林27.6万亩、造林19.3万亩，新建森林公园2个、湿地公园2个，建成乡村绿化美化示范村115条。矿山石场治理复绿49公顷。完成龙塘、石角电子废弃物综合整治二、三期工程。建成横荷应急生活垃圾填埋场工程。

一年来，我们抓民生、促和谐，共建共享幸福清远得到彰显

民生保障更加有力。民生实事全面完成。全市民生支出266.9亿元,占一般公共预算支出78.2%。稳步提高城乡低保、特困人员、医疗救助等底线民生保障水平。新增城镇就业4.4万人，失业率控制在2.38%，劳动技能培训 1.37万人次。社保参保773.4万人次。完成搭建市属国企住房租赁平台，分配政府投资公租房5098套，发放城镇住房保障家庭租赁补贴1451户，完成农村危房改造8837户。

公共服务稳步提升。在粤东西北率先实现省推进教育现代化先进县（市、区）全覆盖。学位供给加快，市中心区域新增1.3万个、全市新增2.1万个义务教育阶段学位，全面消除56人以上大班额。成为现代学徒制全国试点单位。创建全国校园足球特色学校15所。引进广东金融学院。大力推进卫生强市、健康清远建设，医药卫生体制改革继续深化，公立医院全面取消耗材加成，19个县级公立医院升级项目全部动工，乡镇卫生院标准化完成率97.4%，村卫生站规范化完成率98.3%，县域住院率高于全省2.1个百分点。山区、镇村教师和医务人员补助标准稳步提升。基本实现基层综合文化服务中心全覆盖，开展文化惠民千村行、清远马拉松等系列惠民文体活动。

城市环境持续改善。积极谋划中心城区重点片区建设。燕湖新城建设稳步推进，“四馆”动工建设，院南路、院东路建成使用，完成奥体中心、南岸公园等项目规划，优化一江两岸景观。新一轮创文全面启动，广泛开展蓝丝带、文明劝导等活动，大力推进“厕所革命”，突出整治环境卫生、农贸市场、交通秩序等问题，整治老旧小区9个、内街背巷289条，修缮市政道路14.6万平方米，拆除乱搭乱建24.3万平方米。中心城区全面开展集中屠宰、冷链配送、生鲜上市。加强交通拥堵治理，整治14个交通拥堵点，优化120个交通路口信号灯，投放新能源公交车108辆，新增公交300班次。

安全生产形势总体稳定。坚持把安全生产摆在突出位置，开展安全生产百日攻坚。市政府逐一与各县（市、区）、市直相关部门梳理、落实安全生产工作。出台安全生产改革发展、举报奖励等制度。推动明查暗访和交叉检查，深入开展消防、交通运输、危险化学品等专项整治，严厉打击违法行为，消防违法方面拘留26人、安全生产违法方面拘留31人。设立1亿元安全生产专项资金，加快安全隐患点整治，挂牌督办重点安全隐患34个，完成2300公里生命安全防护工程建设。成功应对“山竹”超强台风。全市安全生产事故起数、死亡人数、受伤人数分别下降39.8%、19.9%和41%。

一年来，我们抓作风、强法治，政府自身建设有新成效

法治政府扎实推进。自觉接受市人大及其常委会法律监督，主动接受市政协民主监督，办理人大议案建议129件，政协提案433件。健全政府顾问制度，加强重大决策法律咨询。完善城乡四级公共法律服务体系。行政机关出庭应诉率居全省前列。推进行政执法公示三项制度，首次开展行政执法案卷评查工作。主动公开各类政务信息13万余条。

作风建设深入开展。深入学习宣传贯彻习近平新时代中国特色社会主义思想、习近平总书记参加广东代表团审议及视察广东重要讲话精神，树牢“四个意识”，坚定“四个自信”，坚决做到“两个维护”。坚决全面彻底肃清李嘉、万庆良恶劣影响。认真履行全面从严治党主体责任和党风廉政建设责任。狠抓队伍作风建设，严格落实中央“八项规定”，坚决整治“四风”。深入开展“正风”行动、“公述民评”等活动。推进审计全覆盖。

社会大局和谐稳定。圆满完成改革开放40周年重大专项警卫安保任务。“平安清远”建设加快。全面开展扫黑除恶专项斗争，打掉涉黑涉恶团伙145个。深入推进社会治安综合治理、全民禁毒、“扫黄打非”，治安环境持续向好。落实领导包案、定期接访等制度。城乡社区治理改革、村民自治试点有序推进。食品药品形势稳定向好。粮食保障能力有效提升。统计、民族、宗教、侨务、港澳、人防、国防动员、方志、档案、气象、水文、地震、慈善、妇女儿童、残疾人等工作取得新成绩。

这些成绩的取得，是省委、省政府和市委正确领导的结果，是全市人民和社会各界凝心聚力团结奋斗的结果，是广州等兄弟市大力支持的结果。在此，我代表市人民政府，向全市人民，向驻清部队和各级驻清单位，向所有关心支持参与清远建设发展的各界人士，致以崇高的敬意和衷心的感谢！

回顾一年工作，我们也清醒地认识到，当前清远发展仍然面临不少困难和问题：一是发展不足与不平衡、不协调问题并存。经济总量不大，重大项目不多；南北部之间、各县（市、区）之间，与珠三角地区的发展差距有拉大趋势，城乡二元结构问题突出。二是经济运行结构性矛盾比较突出。传统高耗能产业占比仍然较大，新动能不足，产业总体水平不高；房地产投资占比偏大，实体经济面临较多困难；新增税源不多，收支矛盾突出；外贸进、出口商品结构单一；消费新动力不足，供给质量有待提高。三是要素制约趋紧、使用效益不高。大批产业、城建、交通、民生等项目受政策、土地、资金制约影响，闲置用地、低效用地量大，沉淀资金、绩效管理亟待加强。四是环保、安全生产压力较大。环保、安全生产基础设施不完善，监管能力有待加强，责任落实还不到位，大气污染防治、水环境保护、城市生活垃圾处置、安全生产等形势较严峻。五是基本公共服务水平总体不高。教育、医疗、文化等优质资源供给不足，民生保障水平有待提升。六是政府部门作风建设有待加强，服务效能仍需不断提升。个别部门、少数干部缺乏主动作为、敢于担当、攻坚克难精神，工作不细不实。营商环境、城市管理等有待提升。对此，我们将采取更加有效的措施切实解决。

2019年工作安排

2019年是新中国成立70周年，是深入学习贯彻习近平总书记视察广东重要讲话精神、决胜第一个百年目标的关键之年。国内经济和政策环境总体稳定，“一带一路”扎实推进，粤港澳大湾区建设提速，省“一核一带一区”区域发展格局加快构建，广清一体化高质量推进，一批大项目和平台建设加快，特别是，习近平总书记亲临视察和重要讲话极大鼓舞了清远干部群众，为清远的改革发展标定了航向，这些都为我市高质量发展提供了动力和保障。与此同时，国内外经济下行压力加大、国际贸易保护主义、单边主义，中美贸易摩擦、房地产市场等不确定因素较多，风险和挑战更加复杂。我们必须认清形势、坚定信心、把握机遇、主动作为，全力推进经济社会高质量发展取得新成效。

今年政府工作的总体要求是：以习近平新时代中国特色社会主义思想为指导，全面贯彻党的十九大和十九届二中、三中全会以及中央经济工作会议精神，深入学习贯彻习近平总书记视察广东重要讲话和对广东一系列重要指示批示精神，认真落实省委十二届四次、五次、六次全会和市委七届七次、八次全会部署，坚持稳中求进工作总基调，坚持新发展理念，坚持推动高质量发展，深入实施创新驱动、乡村振兴、广清一体化，努力探索破解城乡二元结构，大力支持实体经济发展，全力筑牢粤北生态屏障，继续打好三大攻坚战，着力加快“入珠融湾”进程，努力保持经济社会平稳健康发展，为全面建成小康社会收官打下决定性基础，以优异成绩庆祝新中国成立70周年。

2019年经济社会发展主要预期目标是：地区生产总值增长6%左右，固定资产投资增长9%左右，社会消费品零售总额增长9.5%左右，进出口总额保持正增长，地方一般预算收入增长7%左右，城镇登记失业率控制在3%以内，居民人均可支配收入增长略高于经济增长，居民消费价格涨幅控制在3%以内；社会保障、资源环境保护、节能减排及安全生产完成省定目标。

为实现上述目标，我们将重点做好以下工作：

一、以深化广清一体化为抓手，全面对接粤港澳大湾区，带动全市区域协调发展

坚持交通先行。以交通互通为先导，完善推进广清交通一体化，加快融入大湾区1小时经济生活圈，加快打造粤东西北“融湾”先行市。实施中心区域道路交通三年行动计划，力争再完成一批道路项目，逐步打通一批断头路；推动佛清从高速清远段、广连高速、二广高速与清连高速连接线等加快建设，确保汕湛高速清云段建成通车，加快打造环城高速，推动主干道路与高速公路对接。完成广清城轨一期建设，全力推动二期动工。完成北江航道扩能升级项目主体工程，清远枢纽、白石窑枢纽二线船闸、飞来峡枢纽二、三线船闸建成投入使用。加快太石路、广清大道、东环快速南延等对接道路前期工作，力争项目尽早动工。积极推动广清永高铁、广清地铁、佛江高速北延线项目纳入大湾区综合交通规划。进一步推进两市公交共建共享。全力推进国省道项目建设，力争新启动160公里、完成50公里新改建。完成编制清远港总体规划，推进大宗货物绿色运输北江示范项目建设。保持治超执法高压态势。

深化产业协同融合发展。抢抓粤港澳大湾区建设机遇，积极参与大湾区产业分工。修编广清一体化产业专项规划。推进广清产业园A区建设，着力建设国家产城融合示范区，力争投（试）产项目达46个；加快B区建设，协调推动广州开发区优惠政策向其覆盖，打造广州知识城协同发展功能区。加快把原两德合作区上升为广清特别合作区。加快各产业集聚平台建设。积极探索“广州总部+清远基地”“广州创造+清远制造”“广州孵化+清远加速”等合作模式，形成梯级递进的产业布局。加强招商引资和全市招商统筹，建立广州、深圳等重点地区招商小分队，强化重大项目招商，举办面向民企、国企、高新企业等招商推介活动。继续推进帮扶引进20亿元、10亿元大项目和产业集群。强化签约项目跟踪落地。加强项目履约监管。完成省定梯度转移珠三角项目和投资任务。力争全市园区工业增加值、全口径税收均增长15%以上。积极高质量承接广州非中心城市功能，规划建设广清空港现代物流产业新城，深化与广州空港经济区功能规划对接，共同打造国际化现代供应链基地，加快广清农业众创空间二期建设，继续推进中英健康城项目。

加快北部生态发展区建设。落实省“一核一带一区”战略部署，充分发挥生态优势，建好“北屏障”，探索加快把绿水青山变成金山银山，拓展发展空间、增强发展后劲。配合做好粤北生态特别保护区建设，开展南岭生态旅游公路规划研究。推进连阳四县（市）国家重点生态功能区建设。继续培育壮大绿色低碳新型工业、特色农林、绿色食品、文化生态旅游和清洁能源等产业；支持民族工业园中小企业生产、双创基地建设，大力发展食品、农产品深加工；支持连山创建“国家有机产品认证示范区”、连南创建“国家级出口食品农产品质量安全示范区”。大力发展林业新兴产业，力争成为全省首个森林碳汇规模化交易市。完成德建水库工程建设。加快二广高速连贺支线、韶贺高速等前期工作。积极向上级争取扶持政策，推动民族地区加快发展。

更好发挥重点项目带动作用。加强项目统筹和要素保障，全力推进重点项目建设，促投资、打基础。安排省、市重点项目161项，年度计划投资297.6亿元。协调推进清远长隆及周边配套设施建设，力争清晖南延伸段、森林大道建成通车。加快省级职教基地建设，确保五校秋季开学。加快推动“四馆”、奥体中心、广东金融学院清远校区建设。力争磁浮旅游专线试运行。推动英德通用机场、连州支线机场加快前期工作。启动潖江蓄滞洪区建设。推动腾讯华南云计算基地、苏宁华南智慧产业园、南部物流枢纽、金发科技等重点产业项目加快建设。

二、坚持转型升级，大力发展实体经济、民营经济，筑牢高质量发展基石

扶持民营经济加快发展。落实中央、省促进民营经济、实体经济发展的各项政策，出台促进民营经济发展措施，在降低企业生产经营成本、破解融资难等方面精准施策，以更有力举措推动我市民营经济实现新发展。鼓励园区建设和使用标准厂房，力争建设标准厂房80万平方米以上，降低企业建设成本，促进产业集聚。制定先进制造业高技术企业扶优计划，提升优质重点企业综合竞争力。完善促进总部经济发展措施，进一步强化总部企业扶持。加快推动企业上市步伐。加强要素保障，鼓励支持企业开展技改、智能化改造和增资扩产，放宽技改普惠政策享受范围，力争推动120家以上企业开展技改。引导金融机构加大实体经济投放力度，设立小微企业转贷基金，推进政策性融资担保业务开展，促进地方农商行（农信社）业务回归本源，支持本地经济发展，着力缓解企业融资难融资贵问题。支持民营企业人才引进。畅通沟通协调机制，及时协调解决企业的问题。

加快建设区域创新中心。主动对接“广深港澳”科技创新走廊，推动高新区扩容提质，争创创新型特色园区，力争进入全国高新区前100位。加强高新企业培育，力争高新技术企业达到260家。推动华南863、华大健康等双创平台加快发展，力争把天安智谷打造成国家级孵化器。鼓励各县（市、区）、园区、企业兴办各类孵化器、加速器，引导更多科技成果到清远转化。支持企业建立技术中心、工程中心、重点实验室、博士后工作站等研发机构，力争新增省级新型研发机构2家以上。加大创新人才支持力度，鼓励企业开展产学研合作，大力引进“扬帆计划”“起航计划”等创新创业团队。推动国家知识产权试点城市建设，力争专利授权量、发明专利授权量、行业标准制（修）订量均增长15%以上。加强质量强市建设，深入推进质量提升行动。着力培育标杆电商企业。完成市属国企改革重组，支持国企做强做大。

大力发展全域旅游。统筹推进全域旅游示范区建设，积极谋划建设北江旅游带，加快打造具有主题公园特色的国际化旅游城市。编制全域旅游发展总体规划、公路规划，指导县域和旅游资源丰富的乡镇编制旅游规划，以高质量规划引领全域旅游开发建设。规划推动特色旅游小镇建设。大力发展乡村旅游、民宿经济、农家乐。推进智慧旅游、“旅游+”发展。全面策划推介清远旅游产品，加强城市旅游联盟、联动，加快完善旅游厕所、标识、交通等公共配套，打造特色购物、休闲、美食街和旅游手信，吸引更多游客过夜，拉动旅游消费。推动旅游项目招商。扶持旅游龙头企业发展，鼓励创建国家4A、5A级景区。打造“清远48小时旅游目的地”。

三、以综合示范区为抓手，加快推动乡村振兴发展，积极探索破解城乡二元结构

创新乡村振兴体制机制。坚持农业农村优先发展，建立健全政策体系，引导人才、资金、技术等要素资源“上山下乡”。推进全国农村改革试验区、省乡村振兴综合改革试点等建设，深化“三个重心下移、三个整合、四个致富、五个梯度创建、六个留下”，筑牢乡村振兴发展基础。推进农村耕地整合治理，加快解决承包地碎片化问题。完善涉农资金统筹整合长效机制。稳步推进农村集体产权制度改革。健全规范农村集体经济组织。大力支持地方农商行（农信社）发展，强化普惠金融服务“三农”能力。提升农村金融服务水平，开展特色农业担保基金业务，拓展农村农业险种等。加强“三农”人才培养引进，推进“新乡贤”工程，强化乡村振兴人才支撑。

探索打造综合示范区。落实习近平总书记“二十字”要求，加快打造乡村振兴综合示范区，高质量走好乡村振兴路。出台示范区试点建设方案，启动第一批、力争建成2-3个乡村振兴综合示范区。打响连樟村区域特色品牌，将连樟村打造成为全国农村基层党建、精准扶贫和美丽乡村建设示范点，将连江口镇打造成为全国乡村振兴发展和特色旅游示范镇。加快推进现有4个省级现代农业产业园建设，继续做好申报创建工作，力争实现县级全覆盖，英德、连州力争2个以上。力争把英德红茶、清远鸡打造成国家级产业园区。继续推进农业公园、田园综合体建设。

突出抓好产业振兴。结合清远特色和产业发展，培育引进农业龙头企业，加快项目落地、产业增效，力争新增省级农业龙头企业5家。大力培育发展新型农业经营主体，力争农民合作社、家庭农场分别新增100家和1000家。创建农业产业联合体。扎实推进“3个三工程”，力争新增茶叶3万亩、柑橘5万亩、清远鸡1700万只。推进农村一二三产业融合发展。实施农业品牌提升行动，加大特色农产品推介，强化市场对接，推动农村电商加快发展，落实清新、英德电商进村示范项目建设，努力把英德电商园打造成全国农产品展销电商示范园。建设清远智慧农业研究院。筹办好第十五届中国茶业经济年会。打造市级“粤菜师傅”培训基地。

推进美丽乡村建设。实施“美丽乡村2025”行动计划，推动“千村示范、万村整治”工程，优先在沿交通线、旅游景区、城市郊区重点推进，力争完成90%以上自然村人居环境综合整治，60%以上非贫困村创建成为美丽乡村，着力打造一批省级示范县、镇、村。力争全市村庄规划编制覆盖率达到50%。加强乡村文明建设，推动古村落、古驿道开发和保护。加快“四好农村路”建设，力争完成农村公路新硬化836公里、中小河流整治126.9公里、农网升级改造投资4.8亿元。

四、弘扬改革创新精神，着力破解一批关键制约问题，再创清远发展新优势

营造更优发展环境。认真梳理“痛点”“堵点”，精简审批流程，提高办事效率，进一步优化营商环境，真正让企业和群众感受到政府办事更加便捷高效。推进相对集中行政许可权试点，加快商事制度、“证照分离”等改革，推动“照后减证”，建立承诺制和“容缺受理”制度，力争企业开办时间压减至3.5个工作日以内，投资项目办理期限压减至5个工作日以内，工程建设项目审批时限压减至100个工作日以内。继续推进强县放权。动态调整权责清单、中介服务事项清单，规范提升中介超市服务效率。加快“数字政府”建设。加强社会诚信建设，建立健全信用信息运用机制。做好第四次全国经济普查。

继续深化供给侧结构性改革。落实“巩固、增强、提升、畅通”八字方针。全力防范金融风险，加强政府债务管理，有序化解政府隐性债务，提升财政资金使用绩效，进一步压降农合机构（农商行）不良贷款率，严厉打击非法集资行为。加强房地产市场监管服务，合理控制节奏，稳定市场预期，培育发展住房租赁市场，促进房地产市场平稳健康发展。落实好各项降费减税政策措施，力争为企业降成本37.1亿元。从严做好产能置换，严防“地条钢”死灰复燃。力争全面完成陶瓷企业“煤改气”。继续推进中心城区商业网点规划建设。加快各项补短板工作。

破解土地制约瓶颈。坚持集约节约用地，精准高效配置土地资源，用好增量、盘活存量。出台国有建设用地留用地流转、提高工业用地利用效率等政策，把好项目用地效益关，突出亩产效益。依法分类处置，全力推进批而未供和闲置土地“去库存”。建立批后监管与年度计划指标挂钩奖惩机制。加快垦造水田。稳步推进农村土地拆旧复垦。有序推进“三旧”改造。整治违法违规用地，严厉打击矿产资源违法行为。进一步提升用地审批效率。

强化安全综合应急管理。坚持安全发展理念，坚守安全生产红线，力争通过一个时期的努力，有效扭转安全生产被动局面。巩固“百日攻坚”成果。实施安全生产三年行动计划。进一步落实安全生产“一岗双责”，压实企业主体责任；强化重点领域、重点区域安全生产专项整治。坚持明查暗访和异地交叉检查常态化；坚持严格执法、重典治乱。加快整改安全隐患。深入推进平安北江建设。加强应急救援队伍和装备建设，整合镇村各类“站”“员”，落实网格排查职能。大力开展安全教育“七进”活动。探索完善安全生产长效机制。

五、加强城乡基础设施建设，提升城市发展内涵、形象与价值，打造美丽舒适宜居城市

全面提升城市功能品质。不断完善城市功能，塑造特色城市风貌，彰显城市文化，让市民生活更便捷舒适。制定城市建设三年行动计划，实施住宅用地三年滚动供应计划，加快建设一江两岸，加强两岸景观布局，推进南、北岸公园等建设，规划完善环北江休闲绿道；推动城市微改造。统筹两区一体化发展，加快推进旅游大道、清远西部一级公路建设。推动中以科技小镇、梦享谷等加快规划建设。优化市区4个高速出入口整体风貌。推动各县（市、区）中心城镇提质建设。指导推进乡镇规划编制。加快通信、电力基础设施建设，力争光纤入户率91%以上，移动宽带普及率87%以上；推动220千伏白庙、汤塘、连州输变电等电网工程建设。

加大文明城市创建力度。坚持把创文作为“一号民心工程”，高标准做好年度各项创建工作。加快完善创文长效机制。实施八项提升行动，突出“四零一围+点位”，重点抓好环境卫生、农贸市场、交通违章整治，大力发展志愿者、义工、社工等公益事业，引导提升市民文明素养。统筹推进创建全国双拥、绿化模范城市、全国禁毒示范城市，巩固创卫创园成果。继续加强交通拥堵治理，推进城市慢行系统、人行天桥规划建设，开展堵点整治改造，加快智能交通建设；加大公交车投放力度。压实网格包干、“门前三包”等责任，提高城市精细化管理水平。

着力解决垃圾处理问题。坚持科学论证，加大科普宣传，强化公众参与，实施生活垃圾集中处理生态补偿，加快推进市区生活垃圾焚烧发电项目规划建设，编制中心城区环卫设施专项规划。完成67个镇级简易垃圾填埋场整治，规范“一镇一站”“一村一点”运作。积极试点应用新技术，推动垃圾减量化、资源化、无害化处理。探索农村生活垃圾治理市场化。推进垃圾分类减量和一般镇区垃圾收费。规范建筑垃圾处置管理。

六、以生态发展区功能定位为引领，全面加强环境保护和治理，把绿色生态打造成清远的核心竞争力

推动空气质量持续改善。继续开展大气污染防治攻坚，系统整治空气污染源，让群众呼吸更清新的空气。加强工地、道路、矿山、码头等扬尘治理。加强露天焚烧和烟花爆竹燃放管理。强化“散乱污”企业（场所）污染治理。完成淘汰落后产能VOCs企业。加快整治生物质成型燃料锅炉，淘汰10蒸吨及以下工业燃煤锅炉。推动充电桩建设，推广应用新能源。

强化水环境保护。抓好滃江流域、大燕河、乐排河、漫水河等重点流域综合整治，确保水质持续改善。全面落实河长（湖长）制，加强河流环境巡查整治。建立河道保洁长效机制，深入开展“五清”专项行动。加强工业集聚区污水集中处理设施建设，推进港口和船舶污染防治。加快15个省级断面水质自动监测站建设。

推进环保督察整改到位。完成澜水河、龙沥大排坑城市建成区段黑臭水体整治。完成县级饮用水源地环境问题整治。加快整县推进污水处理设施建设，力争新增管网160公里，改造旧管网30公里，实现镇级污水处理设施全覆盖。严防禁养区内畜禽养殖反弹，提高畜禽养殖废弃物资源化利用。推进危险废物处置设施建设，严厉打击非法转移倾倒固废行为。

持续推进生态建设。统筹山水林田湖草系统治理。扎实推进新一轮绿化广东大行动，加快林业重点生态工程建设，积极创建国家森林城市，大力推进森林进城、森林围城、森林美城，推进路边、水边、城边绿化，力争新建2个森林公园、1个湿地公园、1个森林小镇和一批绿美古树乡村，加快笔架山、南峡山森林公园等项目建设。推进森林消防机场建设。继续深化国有林场改革。抓好矿山石场治理复绿。强化土壤污染管控和生态修复。

七、加快补齐民生短板，切实保障和改善民生，让群众的日子越过越好

努力提供更多优质公共服务。从人民群众关心的事情做起，一件一件办好民生工作。全力抓好十件民生实事落实。力争创建成为省推进教育现代化先进市。持续加大学位供给。加强农村义务教育寄宿制学校建设管理。推动城镇住宅小区落实配套学校建设。积极引进国内名校到清远办学。推进现代学徒制试点。进一步推进卫生强市、健康清远建设，深化医药卫生体制综合改革，推动“互联网+医疗健康”建设，提升基层医疗卫生服务能力，推进分级诊疗，努力提升全方位、全周期保障群众健康水平。积极谋划推动省运会筹备工作，加快公共文化体育设施建设，举办好清远马拉松、乡村戏曲节、乡镇（街道）篮球赛等群众文体活动。

持续提升保障水平。强化底线民生保障。完善就业创业服务。完善社会保障体系，健全社保扩面征缴长效机制，力推全员足额参保。深化医疗保险改革，健全全民医保制度，完善病种分值结算制度，推进异地联网就医直接结算。加快养老服务体系建设，优化城乡社区居家养老服务，加快市福利院老年公寓建设。落实保障房供给政策。保障和维护好劳动者合法权益。

全力抓好脱贫致富。对标2020年全面脱贫总要求，集中力量啃硬骨头，坚决打赢脱贫攻坚战。深入实施脱贫攻坚三年行动计划，突出抓好产业扶贫，深入开展“百企扶百村”，引导企业投资建设就业扶贫基地和“扶贫车间”，增强“造血”功能。抓好政策兜底保障，重点解决好“两不愁三保障”。加快补齐贫困村基础短板。加强扶贫资金管理。

营造共建共治共享社会新格局。深入开展扫黑除恶专项斗争，依法严惩涉黑涉恶违法犯罪，打掉“保护伞”。坚决打赢全民禁毒攻坚战。深化“平安清远”建设。加快社区治理改革。加强严重精神障碍患者救治救助。培育发挥社会组织作用。做好信访维稳、“扫黄打非”、打假、打传、打私等工作。强化食品药品安全综合治理。加强粮食安全保障。继续做好统计、民族、宗教、外事、侨务、港澳、人防、国防动员、档案、方志、气象、水文、慈善、妇女儿童、残疾人等工作。

八、切实加强政府自身建设，进一步强化执行、优化服务、提升效能，建设人民满意政府

落实全面从严治党要求。严格落实党建主责主业，切实加强政府系统党组织建设。深入学习贯彻习近平总书记视察广东及系列重要讲话精神，进一步树牢“四个意识”，坚定“四个自信”，坚决做到“两个维护”。落实中央八项规定精神。推进中央巡视反馈意见整改。认真履行党风廉政责任制，坚持标本兼治，保持惩腐高压态势。发挥审计监督和保障作用。

提升依法行政能力。坚持党委决策、政府落实。自觉接受人大及其常委会、人民政协的监督，坚持重大事项主动向人大报告、向政协通报，高质量办好人大代表建议和政协委员提案。主动听取社会各界意见建议。深入推进科学立法、民主立法。发挥好政府法律顾问作用。全面落实行政执法责任制，严格公正文明执法，坚持行政机关负责人出庭应诉。深入推进政务公开。完成机构改革。

狠抓政府系统作风建设。坚持不懈抓作风建设，开展政府系统作风整治行动，抓住关键，突出典型，重点整治，强化干部队伍责任担当和落细落小落具体，坚决创造性地贯彻落实中央、省和市委的决策部署。深入开展“正风”行动、“公述民评”等活动，提升12345服务效能，大力整治不作为、乱作为、慢作为等不良作风，整治形式主义、官僚主义等突出问题。健全容错纠错机制，鼓励干部积极作为、靠前服务，着力构建“亲”“清”新型政商关系。

各位代表，新的一年，新的征程。让我们更加紧密团结在以习近平同志为核心的党中央周围，高举习近平新时代中国特色社会主义思想伟大旗帜，在省委、省政府和市委的正确领导下，牢记嘱托，凝心聚力，砥砺前行，为清远加快振兴发展、全面建成小康社会打下坚实基础，以更加优异的成绩迎接新中国成立70周年！
